# Anticancer effects of crocetin in human esophageal squamous cell carcinoma KYSE-150 cells

**DOI:** 10.3892/ol.2015.2869

**Published:** 2015-01-13

**Authors:** SHENG LI, SHENG JIANG, WEI JIANG, YUE ZHOU, XIU-YIN SHEN, TAO LUO, LING-PING KONG, HUA-QIAO WANG

**Affiliations:** 1 Department of Anatomy and Neurobiology, Zhongshan School of Medicine, Sun Yat-sen University, Guangzhou, Guangdong 510080, P.R. China; 2 Department of Cardiothoracic Surgery, Shantou Central Hospital, Affiliated Shantou Hospital of Sun Yat-sen University, Shantou, Guangdong 515000, P.R. China

**Keywords:** esophageal cancer, crocetin, KYSE-150 cells, apoptosis, cell cycle

## Abstract

Crocetin is the main pharmacologically-active component of saffron and has been considered as a promising candidate for cancer chemoprevention. The purpose of the present study was to investigate the anticancer effects of crocetin and the possible mechanisms of these properties in the esophageal squamous cell carcinoma cell line KYSE-150. The KYSE-150 cells were cultured in Dulbecco’s modified Eagle’s medium and incubated with 0, 12.5, 25, 50, 100 or 200 μmol/l crocetin for 48 h. Cell proliferation was measured using an MTT assay. Hoechst 33258 staining and observation under fluorescent microscopy were used to analyze the proapoptotic effects of crocetin. The migration rate was assessed by a wound-healing assay. The cell cycle distribution was analyzed using flow cytometry analysis subsequent to propidium iodide staining. The expression of B-cell lymphoma-2-associated X protein (Bax) and cleaved caspase 3 was determined by western blot analysis. It was found that treatment of KYSE-150 cells with crocetin for 48 h significantly inhibited the proliferation of the cells in a concentration-dependent manner, and the inhibition of proliferation was associated with S phase arrest. Crocetin was also found to induce morphological changes and cell apoptosis in a dose-dependent manner through increased expression of proapoptotic Bax and activated caspase 3. In addition, crocetin suppressed the migration of KYSE-150 cells. The present study provides evidence that crocetin exerts a prominent chemopreventive effect against esophageal cancer through the inhibition of cell proliferation, migration and induction of apoptosis. These findings reveal that crocetin may be considered to be a promising future chemotherapeutic agent for esophageal cancer therapy.

## Introduction

Esophageal cancer is one of the eight most common cancers and is the sixth leading cause of global cancer mortality. An estimate indicates that there were 482,300 novel esophageal cancer cases and 406,800 mortalities due to esophageal cancer in 2008 worldwide (1). The major histological subtypes of esophageal cancer are adenocarcinoma (AC) and squamous cell carcinoma (SCC) (2). There is a high prevalence of AC in Europe, while SCC is the dominant form in Asia (1). The prevalence of esophageal SSC (ESSC) has risen in developing countries, particularly in China (2–4). A notable belt of esophageal cancer occurrence, primarily SCC, extends from Northeast China to the Middle East (3). The high incidence of ESCC is associated with smoking, obesity, consumption of hot beverages and red meat, a high alcohol intake, and a low intake of fresh vegetables and fruit (4). Furthermore, early diagnosis of esophageal cancer remains to be a challenge in clinical practice as early esophageal cancer exhibits no characteristic clinical manifestations and there are no effective screening tools (5). Therefore, the majority of patients are at an advanced stage of disease at the time of diagnosis, and the carcinoma has already metastasized. Clinically, the mainstays of treatment for esophageal cancer include surgical resection, radiation therapy and chemotherapy (6,7). However, chemotherapy and radiotherapy demonstrate acute and chronic toxicities, which often results in not only a cessation of therapy, but also a decrease in the quality life (8,9). For these reasons, esophageal cancer exhibits a poor prognosis and the five-year survival rate, subsequent to diagnosis, is <13% (10). Therefore, novel therapeutic alternatives or agents are urgently required for patients with esophageal cancer.

Currently, increasing numbers of studies investigate plants or herbs for antitumor effects (11–13), as they are generally safe and exhibit no or low toxicity. Saffron is the flower of *Crocus sativus* L. and is generally used as a spice and food colorant. Saffron has also been used as a traditional medicine in China, India and the Arab world since time immemorial. Crocetin, the major component of saffron, is a low molecular weight carotenoid compound (14). Numerous studies have been performed to indicate the medicinal properties of crocetin, including antioxidative (15), antihypertensive (16), antithrombotic (17), anti-inflammatory (18), cardioprotective (19), hepatoprotective (20) and neuroprotective (21) effects. Crocetin also exhibits anticancer and antitumor properties. Numerous studies have reported that crocetin exhibits an inhibitory effect on cell proliferation and cytotoxicity, which has been detected in several malignant cell lines, including human gastric (22), colon (23) and breast (24) cancer cells, and in *in vitro* models. In the benzo(a)pyrene-induced lung carcinoma mouse model, crocetin significantly reversed the pathological changes (25). In the 1-methyl-3-nitro-1-nitrosoguanidine-induced gastric cancer rat model, crocetin demonstrated a significant regression of tumor growth in a dose-dependent manner (22). From these studies, it can be observed that crocetin possesses considerable anticancer properties.

Crocetin has exhibited excellent anticancer properties, while the underlying mechanism remains unclear. KYSE-150 cells are an esophageal squamous cell carcinoma cell line and are widely used as an *in vitro* esophageal cancer model to study esophageal cancer. In the present study, the mechanism of the anticancer action of crocetin in the human esophageal squamous carcinoma KYSE-150 cell line was examined by evaluating its antiproliferative, proapoptotic and inhibitory effects on migration. In addition, the intracellular signaling pathway of apoptosis was also investigated.

## Materials and methods

### Reagents

Crocetin (C_20_H_24_O_4_; molecular weight, 328.4) was obtained from MP Biomedicals (Santa Ana, CA, USA). The crocetin was dissolved in dimethyl sulfoxide (DMSO) stored at −20°C and then diluted in medium prior to each experiment. The final DMSO concentration did not exceed 0.1% throughout the study. MTT, Hoechst 33258 and DMSO were purchased from Sigma-Aldrich (St. Louis, MO, USA). Propidium iodide (PI) was obtained from Beijing Dingguo Biotech Co., Ltd (Beijing, China). A bicinchoninic acid (BCA) Protein Assay kit was purchased from Beyotime Institute of Bioengineering (Haimen, Jiangsu, China). Cleaved monoclonal rabbit anti-human caspase 3 antibody (cat. no. 9664) was obtained from Cell Signaling Technology Inc. (Danvers, MA, USA) and polyclonal rabbit anti-human B-cell lymphoma-2-associated X protein (Bax) (cat. no. ab7977) and monoclonal rabbit anti-human β-actin (cat. no. ab179467) antibodies were purchased from Abcam (Cambridge, UK). Horseradish peroxidase-conjugated goat anti-rabbit antibodies were obtained from Wuhan Boster Biological Technology, Ltd. (BA1054-0.5, Wuhan, Hubei, China).

### Cell culture

The esophageal squamous carcinoma KYSE-150 cell line (Japanese Collection of Research Bioresources Cell Bank, Osaka, Japan) was grown in Dulbecco’s modified Eagle’s medium (DMEM) supplemented with 10% fetal bovine serum, 100 units/ml penicillin and 100 μg/ml streptomycin (Gibco Life Technologies, Carlsbad, CA, USA). The cells were cultured under an atmosphere of 5% CO_2_ and 95% air at 37°C.

### Cell proliferation MTT assay

Cell proliferation was measured by an MTT assay as previously described (26). Briefly, the cells were plated in 96-well plates at a density of 1.1×10^4^ cells/well in complete DMEM, and incubated at 37°C. After 24 h, the wells were washed with PBS, and the cells were incubated with 0, 12.5, 25, 50, 100 or 200 μmol/l crocetin for 48 h. The 96-well plate was gently washed with PBS and then MTT was added and left for ~4 h. The resulting formazan was dissolved using DMSO. The product was measured at 570 nm in a microplate reader (Tecan Austria GmbH, Grödig, Austria). All experiments were performed at least in triplicate.

### Migration of KYSE-150 cells in a wound-healing assay

An *in vitro* wound-healing assay was performed to detect the effect of crocetin on cell migration. Briefly, the KYSE-150 cells were seeded in 12-well plates at the density of 1.6×10^5^ cells/well, when the cells had grown to 90% confluency, the cell monolayers were scratch-wounded in a straight line using a 1,000 μl pipette-tip, providing a wound width of 1 mm. Subsequently, the cells were washed three times with PBS to remove the detached cells, and then incubated with 0, 100 or 200 μmol/l crocetin for 48 h at 37°C. To measure cell migration, a phase-contrast inverted microscope (Carl Zeiss AG, Oberkochen, Germany) was used to capture images in four randomly chosen fields within the wounded region at 0, 24 and 48 h. The migration rate was calculated as follows: migration rate (%) = (original width - closure width) / original width × 100%.

### Morphological detection of apoptosis

The cells were seeded in a 12-well plate at a density of 1.2×10^5^ cells/well. Following an overnight attachment period, the cells were treated using 0, 100 or 200 μmol/l crocetin for 48 h. The cells were then incubated with 1 μg/ml Hoechst 33258 for 10 min at 37°C in a humidified atmosphere in the dark, and washed three times with PBS. A fluorescent microscope (Leica Microsystems GmbH, Wetzlar, Germany) was used to evaluate the nuclear morphology of the cells.

### Cell-cycle analysis by flow cytometry

Subsequent to incubation with 0, 100 or 200 μmol/l crocetin for 48 h, the KYSE-150 cells were harvested and fixed overnight in 70% ethanol at 4°C. The fixed cells were washed with PBS then stained with PI (50 μg/ml) for 30 min at 37°C in the dark. The stained cells were assessed using flow cytometry (Beckman Coulter Cell, USA) and analyzed by FlowJo 7.6.5 software (FlowJo, LLC., Ashland, OR, USA).

### Western blot analysis

The KYSE-150 cells were harvested following incubation with 0, 100 or 200 μmol/l crocetin for 48 h. The cells were then lysed in ice-cold lysis buffer (1× PBS; 1% NP40; 0.1% SDS; 5 mm EDTA; 0.5% sodium deoxycholate; 1% PMSF) for 30 min. The homogenate was centrifuged at 14,000 × g for 15 min at 4°C, the supernatant extract was gathered and quantified for protein using the BCA Protein Assay kit. Equal amounts of cell protein were separated by electrophoresis on 12% SDS-PAGE. The protein was then transferred to polyvinylidene difluoride membranes, and the membranes were blocked using 5% bovine serum albumin for 1 h at room temperature, followed by incubation overnight at 4°C with primary antibodies for cleaved caspase 3 (1:2,000), Bax (1:1,000) and β-actin (1:2,000). The membranes were washed three times with Tris-buffered saline (Guangzhou Whiga Biotechnology Co., Ltd., Guangzhou, China) containing 0.05% Tween-20 (Wuhan Boster Biological Technology, Ltd.), and then incubated with horseradish peroxidase conjugated anti-rabbit antibodies for 1 h. The protein bands were visualized using electrochemiluminescence and the band intensity was measured using Image J 1.46r software (National Institutes of Health, Bethesda, MA, USA). Each experiment was repeated at least three times.

### Statistical analysis

All data were reported as the mean ± standard error of the mean and were obtained from at least three independent experiments. The differences in data were analyzed by one-way analysis of variance followed by the least significant difference test, using SPSS 16.0 software (SPSS, Inc., Chicago, IL, USA). P<0.05 was considered to indicate a statistically significant difference.

## Results

### Crocetin inhibits the proliferation of KYSE-15O cells

**Figure 1:**
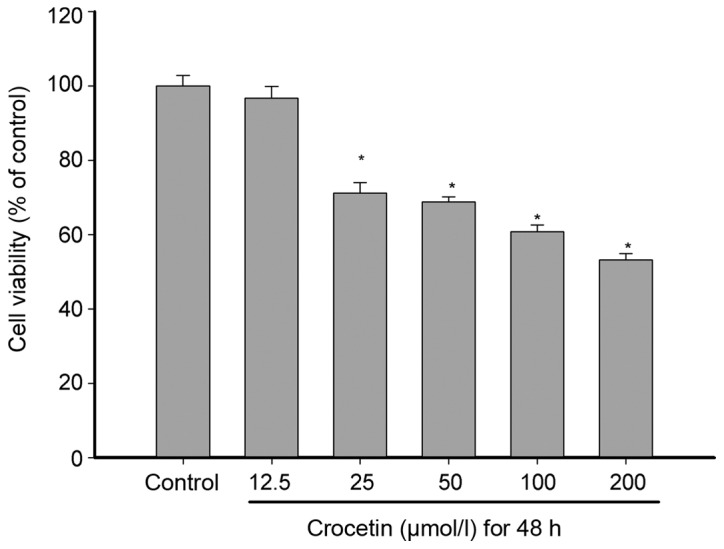
Inhibition of cell proliferation effect of crocetin on KYSE-150 cells detected by an MTT assay. The cells were untreated (control) or treated with 12.5, 25, 50, 100 or 200 μmol/l crocetin for 48 h. Assessment of absorbance at 570 nm revealed a significant decrease in the cell viability of crocetin-treated cells compared with control cells (^*^P<0.05).

An MTT assay was used to measure the inhibition of crocetin on KYSE-150 cell proliferation. As shown in Fig. 1, crocetin inhibited the proliferation of KYSE-150 cells, in a concentration-dependent manner. Cell proliferation was inhibited by all concentrations of crocetin, with the exception of the 12.5 μmol/l group. The cell viability was 96.68, 71.10, 68.76, 60.77 and 53.22% at crocetin concentrations of 12.5, 25, 50, 100 and 200 μmol/l, respectively, revealing a notable inhibition of the proliferation of KYSE-150 cells at higher concentrations.

### Crocetin induces morphological changes in KYSE-150 cells

**Figure 2:**
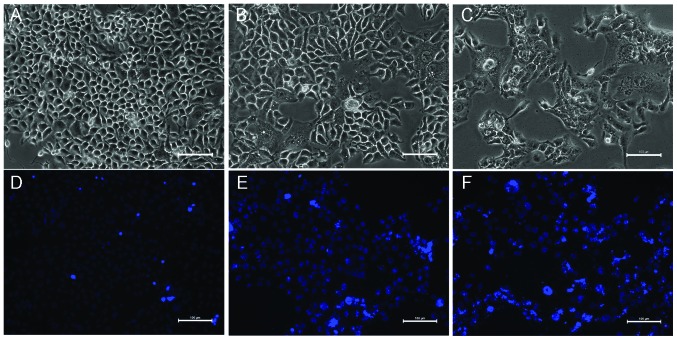
Morphological changes in KYSE-150 cells. The cells were untreated (control) or treated with 100 or 200 μmol/l crocetin for 48 h, followed by incubation with Hoechst 33258 for 10 min to detect apoptosis. The unstained (A) control group and (B) 100 and (C) 200 μmol/l crocetin-treated cells were viewed using light microscopy. The (D) control group and (E) 100 and (F) 200 μmol/l crocetin-treated cells stained with Hoechst 33258 were viewed using a fluorescent microscope. Scale bar, 100 μm.

A morphological method was used to observe the changes in the morphology of the KYSE-150 cells during crocetin-induced cell death. As shown in Fig. 2A–C, normal KYSE-150 cells possess a plump cell body, exhibiting a polygonal shape and distinct cell borders under light microscopy. However, subsequent to 48-h incubation with concentrations of 100 and 200 μmol/l crocetin, the morphology of the KYSE-150 cells changed. The cell number was decreased and the cells became granulated, the cell size reduced, cytoplasmic vacuolar changes occurred and certain cells even lysed or became replaced by debris, and cellular detachment was prominent. This was particularly notable at a 200 μmol/l concentration of crocetin.

Hoechst 33258 is a membrane-permeable DNA dye. Subsequent to staining with Hoechst 33258, the nuclei of the live cells became blue, while the apoptotic cell nucleus clearly showed highly condensed or fragmented chromatin with inhomogeneous blue fluorescence. Briefly, the cells were stained with 1 μg/ml Hoechst 33258 for 10 min following treatment with crocetin. Crocetin was demonstrated to induce apoptosis in KYSE-150 cells after 48 h, particularly at a concentration of 200 μmol/l. As shown in Fig. 2D–F, fluorescence dense particles of the cell nucleus, nuclear chromatin condensation and fragmentation, and the formation of apoptotic bodies were observed in the crocetin-treated cells under fluorescent microscopy. However, no inhomogenous blue fluorescence in the cell nucleus were observed in the control group and only a small number of cells exhibited nuclear chromatin condensation.

### Crocetin inhibits the migration of KYSE-150 cells

**Figure 3:**
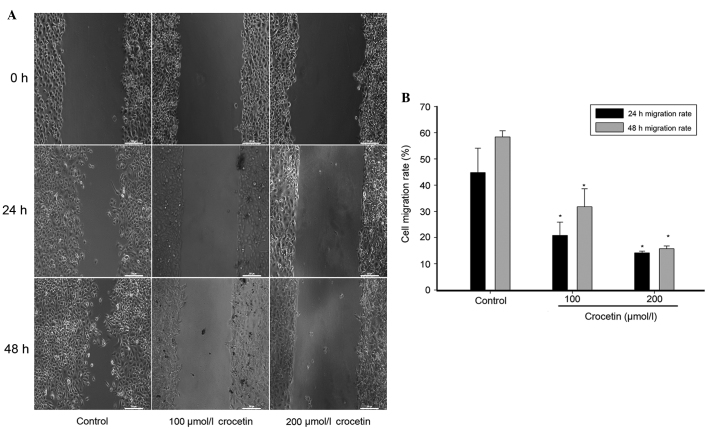
Migration of KYSE-150 cells was determined by a wound-healing assay. (A) Images of the wounded monolayer of KYSE-150 cells captured immediately subsequent to wounding, and after 24 and 48 h. The cells were untreated (control) or treated with 100 or 200 μmol/l crocetin. (B) Cell migration rate of the three groups, calculated as follows: Migration rate (%) = (original width / closure width) - original width × 100%. ^*^P<0.05 vs. untreated group. Scale bar, 100 μm.

A wound healing assay was performed to measure the migration capability of KYSE-150 cells. As shown in Fig. 3, after 48 h the untreated KYSE-150 cells had migrated into the wounded area of the cell monolayer, whereas the migration capability of crocetin-treated cells was significantly reduced, particularly in the group treated with 200 μmol/l crocetin. The migratory rate at 24 h was 44.83% in the control group, and 20.82 and 14.15% in the groups treated with 100 and 200 μmol/l crocetin, respectively. The migratory rate at 48 h was 58.36% in the control group, and 31.78 and 15.71% in the cells treated with 100 and 200 μmol/l crocetin, respectively. A statistically significant difference (P<0.05) was identified between the migratory rates of the crocetin-treated cells and the control, at 24 and 48 h. This indicates that crocetin inhibits the migration of KYSE-150 cells.

### Crocetin induces cell cycle arrest in KYSE-150 cells

To elucidate the underlying mechanism of the inhibitory effect of crocetin on cellular proliferation, the cell cycle distribution in crocetin-treated cells was determined using flow cytometry. As shown in Table I, crocetin significantly increased the number of KYSE-150 cells in the S phase in a concentration-dependent manner. The percentage of cells in the S phase varied between 12.93% in the control group and 23.33% in the group treated with 200 μmol/l crocetin. Concurrently, the number of cells in the G_1_ phase was markedly reduced, indicating that crocetin inhibits the proliferation of KYSE-150 cells by inducing cell cycle arrest in the S phase.

### Crocetin induces the expression of apoptosis-associated proteins

**Figure 4:**
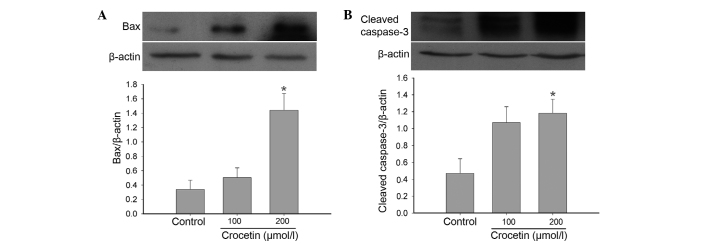
The effect of crocetin on (A) B-cell lymphoma-2-associated X protein (Bax) and (B) cleaved caspase 3 protein expression in KYSE-150 cells was detected by western blot analysis. A statistical comparison was made using analysis of variance followed by a least significant difference test. The gray value demonstrated that treating cells with 200 μmol/l crocetin increased the expression of cleaved caspase 3 and Bax. ^*^P<0.05 vs. untreated group.

To investigate the underlying mechanism of the proapoptotic properties of crocetin, the levels of Bax and cleaved caspase 3 were examined in KYSE-150 cells by western blotting. The levels of Bax and cleaved caspase 3 were markedly increased in crocetin-treated cells compared to the control group (Fig. 4).

## Discussion

In the previous two decades, natural products have been becoming a popular area for studies into chemopreventive and chemotherapeutic agents for the treatment of cancers (11). Crocetin is the main constituent of saffron, and there have been numerous studies into its anticancer and antitumor properties. Several hypotheses for these anticarcinogenic and antitumor effects have been proposed, including inhibition of nucleic acid synthesis, inhibition of free radical chain reactions and the generation of reactive oxygen species through eliminating free radicals, as well as conversion to vitamin A, which enhances carcinogen metabolism (14,27,28). However, the exact mechanism of the anticancer and antitumor effects of crocetin requires additional investigation. Thus, in the present study, the inhibition of cell proliferation and migration and the proapoptotic effects of crocetin on KYSE-150 cells were investigated. To the best of our knowledge, the present study is the first to report crocetin inducing the inhibition of proliferation, proapoptotic effects and the inhibition of migration in the KYSE-150 cell line.

One feature of malignancy is infinite proliferation. Thus, suppression of cell growth has become an important target in cancer therapy. There is a growing body of evidence indicating that crocetin and the analogues of crocetin from various crocus species can inhibit cancer cell proliferation (29). In the present study, it was found that crocetin produced a marked reduction in the proliferation of KYSE-150 cells in a concentration-dependent manner. As it is known that the infinite proliferation of the malignancy is closely associated with the cell cycle regulation (30,31), the cell cycle distribution was detected in the present study in order to explore the underlying mechanism of crocetin. Cell cycle progression is monitored by cell cycle checkpoints that ensure the order and timing of cell cycle transition (32). In addition, the proper replication and segregation of genetic material to daughter cells is crucial. Disorder in cell cycle regulation causes the endless proliferation of cells, leading to cancer (33). Inducing cell cycle arrest has become a key area of antitumor drug development and there are numerous chemopreventive drugs used in the clinic that are based on this principle, such as cisplatin (34). In the present study, treatment with crocetin significantly increased the number of KYSE-150 cells in the S phase. Li *et al* (23) demonstrated that crocetin induced cell cycle arrest in the S phase of SW480 cells through decreased levels of cyclin A and cdk2. However, certain studies have reported that crocetin induces cell cycle arrest in the G_1_ or G_2_ phase (28,29). The variation in cell lines and dosages used in these studies may be responsible for this difference. The exact mechanism of the effects of crocetin requires further investigation.

Apoptosis, or programmed cell death, is a gene-regulated phenomenon, and disequilibrium between cell proliferation and apoptosis is known to cause various diseases, including cancer. Resisting cell death is another notable characteristic of cancer (35). Therefore, the induction of apoptosis in malignant cells is considered to be an important target for the therapy and prevention of cancer (36). The proapoptotic effect of crocetin has been demonstrated in various human tumors, including gastric, colon, breast, liver and pancreatic cancer cells (22–24,28,37). In the present study, following incubation with crocetin for 48 h, the KYSE-150 cells exhibited wide cytoplasmic vacuole-like areas, reduced cytoplasm, cell shrinkage, pyknotic nuclei and the formation of apoptotic bodies, suggesting proapoptotic effects of crocetin. Additionally, crocetin exhibits no toxic effects towards non-malignant cells (23). Overall, this indicates that crocetin meets the requirements of a novel chemotherapy agent that may effectively target cancer cells, whilst exhibiting no or negligible toxicity towards non-malignant cells. Selective induction of apoptosis is one of the major goals of cancer chemotherapy. To investigate the precise mechanism responsible for the selectivity of crocetin, the levels of Bax and cleaved caspase 3 were determined. Sequential activation of caspases plays a central role in the execution phase of cell apoptosis, with caspase 3 being the downstream effector of apoptosis (39,40). Activated caspase 3 is necessary in the mitochondrial and death receptor-mediated cell apoptosis pathways. Bax is located in the cytoplasm and once activated, Bax inserts into the mitochondrial membrane, increasing the membrane permeability and leading to the release of cytochrome *c* (38). Cytochrome *c* then binds with apoptotic protease activating factor-1 and ATP to form an oligomeric apoptosome. The apoptosome binds and cleaves pro-caspase 9, releasing activated caspase 9, which continues to activate caspase 3 and ultimately results in an apoptotic cell (39). In the present study, the levels of cleaved caspase 3 and Bax were significantly increased. Therefore, it was deduced that crocetin exerts its proapoptotic effects by increasing the levels of proapoptotic proteins.

Metastasis is one of the most common features of malignancy, and is characterized by the ability of cancer cells to invade into surrounding tissues, permeate blood or lymphatic vessels and extravasate into a distant environment, which is primarily responsible for the poor prognosis of esophageal cancer (41). It has been reported that >50% of patients with esophageal cancer possess an incurable metastatic disease at the time of diagnosis (42). Therefore, it is imperative to explore the underlying mechanism of metastasis and develop an effective chemopreventive agent to halt the metastasis of esophageal cancer. Numerous studies have revealed that carcinoma cell metastasis demonstrates a close association with the loss of cell-cell adhesion, downregulation of the extracellular matrix, production of chemotactic factors and angiogenesis (43–45). There is a growing body of evidence indicating that crocetin may downregulate matrix metalloproteinases and intercellular adhesion molecule-1, and inhibit angiogenesis (46,47). The present study confirmed that crocetin treatment inhibited KYSE-150 cell migration in a concentration-dependent manner. Although the underlying mechanism of the crocetin-mediated suppression of KYSE-150 cell migration has yet to be elucidated, it can be speculated, based on these aforementioned studies, that crocetin inhibits carcinoma cell migration via multiple pathways rather than a single and specific pathway.

Although the exact mechanism for the anticancer properties of crocetin requires additional investigation, the present results confirmed that crocetin exhibits anticancer properties through three pathways as follows: the inhibition of cell proliferation by blocking the cell cycle progression between S and G_2_ phase; the induction of apoptosis by increasing the activity of the proapoptotic protein Bax and the activation of caspase 3 levels; and the inhibition of carcinoma cell migration. In summary, crocetin may be considered to be a promising chemopreventive agent for the treatment of esophageal cancer.

## Figures and Tables

**Table I:** Effect of different concentrations of crocetin on the cell cycle distribution of KYSE-150 cells.

		Distribution of cells, %		
		
Groups	Crocetin, μmol/l	G_0_/G_1_ phase	S phase	G_2_/M phase
Control	0	78.23±1.32	12.93±0.41	7.03±0.31
Crocetin	100	77.33±0.62	17.73±0.56^a^	4.10±0.29^a^
	200	70.00±0.87^a^	23.33±0.88^a^	6.52±0.20

Cells were untreated (control) or treated with 100 or 200 μmol/l crocetin for 48 h. The cells were then harvested and stained with propidium iodide for flow cytometric analysis. There was a significantly increased number of KYSE-150 cells in the S phase in the groups treated with crocetin compared with the control cells.

a P<0.05 vs. control.
